# Supplementary material for: Understanding and barriers of professional identity formation among current students and recent graduates in nursing and midwifery in low resource settings in two universities: a qualitative study
Source: BMC Nurs. 2024 Mar 1;23:146. doi: 10.1186/s12912-024-01795-2 (PMC10908141; doi:10.1186/s12912-024-01795-2)
Supplement: Supplementary file 1 — Supplementary Material 1 [file 12912_2024_1795_MOESM1_ESM.docx]

## Focus group discussion guide for the Recent graduates

**Duration of interview**: 45-90 minutes

**Introduction:** Hello and welcome to the interview.

My name is …………………………. and I am the moderator for this interview.

Introduce observer/ note-taker……. Who will not actively participate in the discussion but will assist by taking notes.

The topic of discussion is about **Exploring the understanding, perceptions and experiences of professional identity**

The discussion will take between 30-60 minutes. All the information you give is confidential. There are no wrong answers,

We ask for permission to tape-record this session because we do not want to miss your comments. In our research, there will not be any names attached to your views.

My role is to ask specific questions and guide the discussion without putting my views. Feel free to have a lively discussion. It is okay for you not to answer a question you may not be comfortable with.

Let us begin by introducing each other. You are free to use a nickname.

**Nursing/midwifery students**

1. How can you explain professional identity in nursing? (*What is meaningful to you in your work as a nurse? What makes you feel special or unique in your work as a nurse?*)
2. Describe challenges to professional identity formation in nursing? (*What makes you feel frustrated in you work as a nurse?)*
3. Describe barriers to professional identity formation in nursing?
4. Do you think your professional identity has changed throughout your career or stay in the nursing school? Explain why you think your professional identity has changed or Why do you think your professional identity has remained the same?

## Focus group discussion guide for the last year students

**Duration of interview**: 30-60 minutes

**Introduction:** Hello and welcome to the interview.

My name is …………………………. and I am the moderator for this interview.

Introduce observer/ note-taker……. Who will not actively participate in the discussion but will assist by taking notes.

The topic of discussion is about **Exploring the understanding, perceptions and experiences of professional identity**

The discussion will take between 30-60 minutes. All the information you give is confidential. There are no wrong answers,

We ask for permission to tape-record this session because we do not want to miss your comments. In our research, there will not be any names attached to your views.

My role is to ask specific questions and guide the discussion without putting my views. Feel free to have a lively discussion. It is okay for you not to answer a question you may not be comfortable with.

Let us begin by introducing each other. You are free to use a nickname.

**Recent graduates**

1. How can you define Professional Identity in Nursing? (*What is meaningful to you in your work as a nurse? What makes you feel special or unique in your work as a nurse?*)
2. Describe challenges and barriers to professional identity formation? (*What obstacles did you encounter in your workplace in your professional commitment? What makes you feel frustrated in you work as a nurse?)*
3. Identify nurse educator practices that will foster Professional Identity in Nursing.
4. How do you perceive the nursing professional identity?
5. What are some of the experiences in your recent roles and practice which have been instrumental in your realisation of professional identity? (Have you of lately in your practice experienced professional identity and how have you done so?)
6. Do you think your professional identity has changed throughout your career/practice? Explain why you think your professional identity has changed or Why do you think your professional identity has remained the same?
